# Genetic differentiation of the *Schizothorax* species complex (Cyprinidae) in the Nujiang River (upper Salween)

**DOI:** 10.1038/s41598-017-06172-5

**Published:** 2017-07-20

**Authors:** Weitao Chen, Xingjian Yue, Shunping He

**Affiliations:** 1 The Key Laboratory of Aquatic Biodiversity and Conservation of Chinese Academy of Sciences, Institute of Hydrobiology, Chinese Academy of Sciences, Wuhan, Hubei 430072 China; 2 Graduate School of Chinese Academy of Sciences, Beijing, 10001 China; 3 School of Life Sciences, Neijiang Normal University, Neijiang, Sichuan 641100 China

## Abstract

Phenotypically diverse species from recently evolved groups always share allele/haplotype due to insufficient differentiation in the early process. In this study, we performed population genetics analyses using sequences from the mitochondrial cytochrome *b* gene, and two nuclear genes to investigate the genetic differentiation of the closely related *Schizothorax* species complex, comprising a group of alpine fish living in the Nujiang River. The results from both mtDNA and nDNA markers revealed relatively low but pronounced genetic differentiation among the three *Schizothorax* species, i.e., *Schizothorax gongshanensis*, *S*. *lissolabiatus*, and *S*. *nukiangensis*. However, haplotype sharing was frequently occurred among the three species. Divergence time estimation suggested the last glaciation on the Tibetan Plateau (0.075–0.01 Ma) might drive the divergence of the species complex. Gene flow might contribute to the haplotype sharing between *S*. *gongshanensis* and *S*. *lissolabiatus*, and between *S*. *gongshanensis* and *S*. *nukiangensis*, whereas retention of ancestral polymorphisms seemed to be a better explanation of the haplotype sharing between *S*. *lissolabiatus* and *S*. *nukiangensis*. In addition, *S*. *lissolabiatus* populations should obtain more protection in the future because of their low genetic diversity and habitat fragmentation. In summary, our study assesses genetic differentiation among the three closely related *Schizothorax* species and explores the possible driving forces for their differentiation.

## Introduction

In many DNA-based analyses, the genes of different populations and species from recently evolved groups exhibit insufficient differentiation signature in this early process^1, 2^, resulting in allele sharing among phenotypically diverse species. The retention of ancestral polymorphisms and hybridization can both contribute to allele sharing. When an ancestral population harbors a genetic polymorphism, descendant lineages are expected to share polymorphic alleles, reflecting insufficient time to coalescence. However, when pre-mating barriers are not sufficient to hinder local gene flow among young species, these organisms also share genetic polymorphisms in some areas of the genome through hybridization^3^. Even low levels of gene flow can maintain the sharing of polymorphisms across porous species boundaries, and ancestrally polymorphic alleles can reach fixation over time due to natural selection and/or genetic drift^4^.

Schizothoracine (Cyprinidae) fishes, representing the largest and most diverse taxon of the highland ichthyofauna, dominate the torrential mountain streams and plateau lakes of Central Asia, the Himalayas, and peripheral regions of the Tibetan Plateau^5, 6^. The genus *Schizothorax* is the most diversified schizothoracine genus, possessing more than 100 species and subspecies (www.fishbase.org). However, in this genus, the discrepancies between classical taxonomy based on morphology and the molecular phylogeny were frequently observed^7–9^. For example, allele sharing is ubiquitous among different morphological species in the same drainage^7, 9^.

Nujiang River (upper Salween) is an important international river originating from the Tibetan Plateau and flowing through the southwest mountain regions of China. This region has retained a distinct fish fauna, reflecting its complex geological history and landscape diversity^10^. The Nujiang River basin harbors four recognized morphological species/subspecies of the genus *Schizothorax*, *Schizothorax gongshanensis*, *S*. *lissolabiatus*, *S*. *nukiangensis* and *S*. *yunnanensis paoshanensis* ^6, 11, 12^. *S*. *gongshanensis* and *S*. *nukiangensis* are endemic in Nujiang River. *S*. *gongshanensis* occurs only in a small area in the main stem between north Yunnan and east Tibet and in some tributaries in the middle and lower drainage (Figure S1), and *S*. *nukiangensis* is widespread throughout the main stem and large tributaries in Tibet^6, 11–13^. In contrast, *S*. *lissolabiatus* occupies many isolated drainages in Southwest China, e.g., Nujiang River, Lancang River, Red River, and upper Pearl river. However, Yang *et al*. (2012) found that *S*. *lissolabiatus* from the Nujiang River was phylogenetically distinct with *S*. *lissolabiatus* from other drainages^9^, which suggests independent evolutionary history of *S*. *lissolabiatus* in the Nujiang River and rules out the possibility of colonization from other drainages. In the Nujiang River, *S*. *lissolabiatus* only occurs in the tributary of the middle and lower drainage (Figure S1)^6, 11–13^. *Schizothorax yunnanensis paoshanensis* is exclusively observed in the Donghe River basin (Longwang spring and Beimiao reservoir) and Lanzha River in the middle Nujiang River drainage^6, 11–13^. *Schizothorax yunnanensis paoshanensis* and *S*. *yunnanensis yunnanensis* can be distinguished in the length of mouth palpus and distribution^6^. *Schizothorax yunnanensis yunnanensis* only distributes in the Lancang River^6^.

Previous phylogenetic analyses have demonstrated that *S*. *gongshanensis*, *S*. *lissolabiatus*, and *S*. *nukiangensis* cluster into a single lineage with low genetic difference and even share mitochondrial DNA (mtDNA) haplotypes^9, 14^. However, the level of genetic differentiation among the three closely related species remained unresolved. Furthermore, *Schizothorax yunnanensis paoshanensis* generated a sister group with a *Schizothorax* species complex in the Irrawaddy River basin rather than the Nujiang River species complex^9^, *S*. *yunnanensis paoshanensis* was excluded in the present study. Nearly all previous DNA-based studies of the *Schizothorax* species complex in the Nujiang River basin used only mtDNA fragments with limted sample size and did not include nuclear DNA (nDNA) information^9, 13^.

Herein, our aims were to assess genetic differentiation among the three closely related *Schizothorax* species complex and to explore the possible driving forces for their differentiation through dense sampling combining both mtDNA and nDNA markers. In addition, given that cyclical cooling-warming events during the Pleistocene could facilitate population divergence and consequent speciation^15–17^, we also examined whether the Pleistocene climatic oscillations influenced the genetic differentiation of the three closely related species.

## Results

### Sequence information

**Figure 1:**
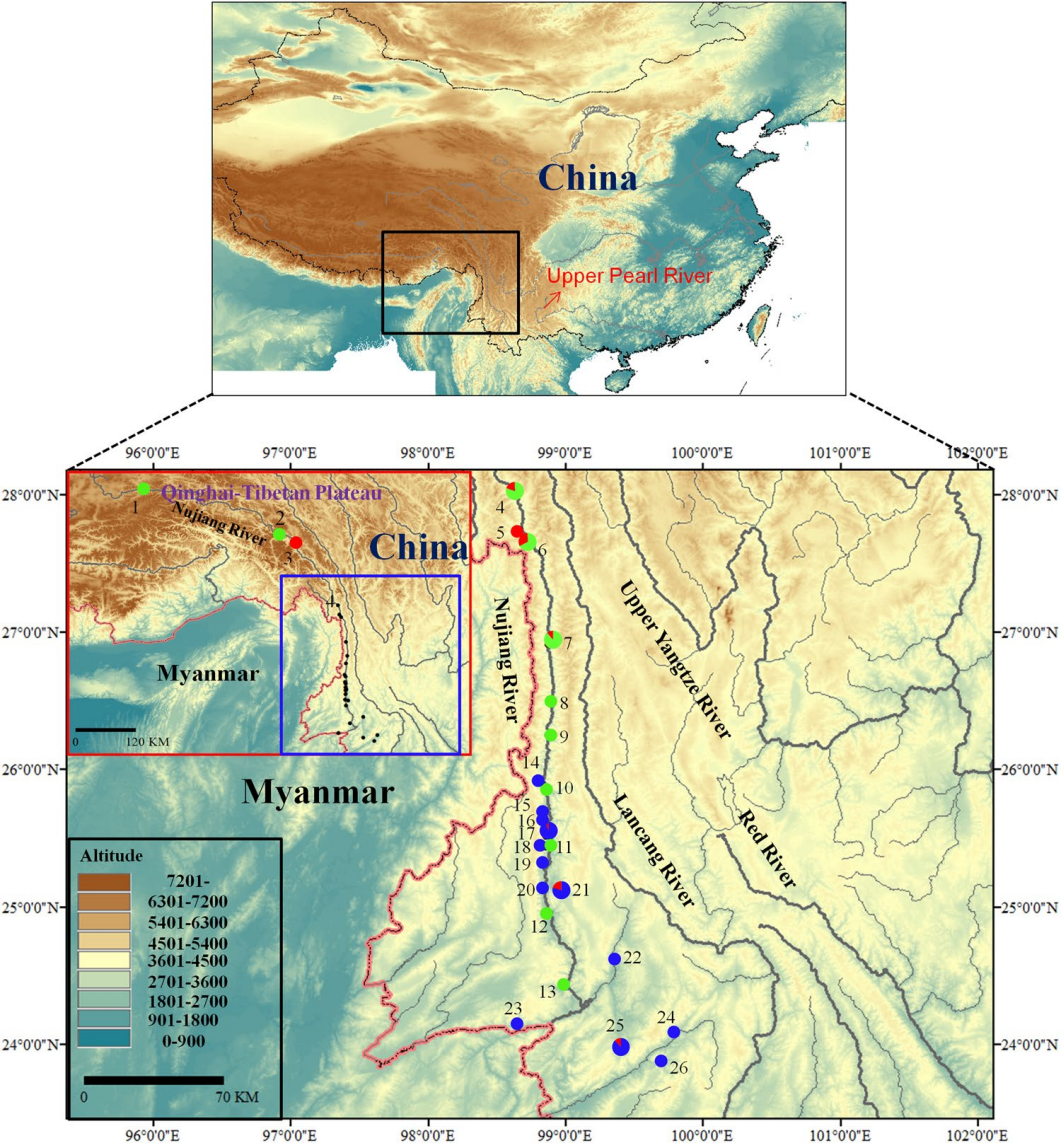
Map of the sampling sites for the *Schizothorax* species complex in the Nujiang River. The site numbers are presented in Table S1. 1–13, main stem sampling sites; 14–26, sampling sites for *S*. *gongshanensis* and *S*. *lissolab1atus* of the tributary in the Nujiang River. Populations are presented as pie-diagrams with slice-size proportional to the frequency of the three species (Red: *S*. *gongshanensis*; blue: *S*. *lissolab1atus*; and green: *S*. *nukiangensis*). Sample sites harbored two species are exhibited with larger pie-diagrams. Map was created in the ArcGIS version 10.1 and modified in Microsoft Office.

We used a total of 447 mitochondrial cytochrome *b* gene (*Cytb*) sequences, including 55 *de novo* sequences, 224 sequences (Genbank nos: KM070647–KM070729) from Chen *et al*.^18^, 151 sequences (Genbank nos: KP796151–KP796154, KP796156–KP796158 and KP796160–KP796168) from Yue *et al*.^13^, and 17 sequences downloaded from NCBI database. The overall *Cytb* sequences came from 26 locations in the Nujiang River basin (Table S1; Fig. 1). The 447 *Cytb* (1063 bp) sequences contained 49 variable sites and 33 parsimony-informative sites. A total of 40 haplotypes were defined from *Cytb* sequences (Table 1).

**Table 1:** mtDNA and nDNA haplotype of the three species in each sampling site. The site numbers are presented in Table S1. n, number of individuals.

Species	Location	n	*Cytb* haplotype	*RAG-1* haplotype	*RAG-2* haplotype
*S*. *gongshanensis*	3	2	H1/H7		
*S*. *gongshanensis*	4	2	H1	R2	R1/R3
*S*. *gongshanensis*	5	36	H1/H7/H12/H39/H40		
*S*. *gongshanensis*	6	14	H1/H5/H6/H7	R2	R1/R2/R3/R5
*S*. *gongshanensis*	7	2	H6/H8		
*S*. *gongshanensis*	17	1	H1	R4/R5	R1/R4
*S*. *gongshanensis*	21	13	H32/H33		
*S*. *gongshanensis*	25	2	H34		
*S*. *lissolab1atus*	14	1	H1		
*S*. *lissolab1atus*	15	17	H1	R4/R6/R7/R10	R1/R6/R9/R10
*S*. *lissolab1atus*	16	2	H32	R4	R5/R11
*S*. *lissolab1atus*	17	15	H1	R6/R7/R8/R9	R1/R2/R4/R7/R8
*S*. *lissolab1atus*	18	20	H32		
*S*. *lissolab1atus*	19	20	H4		
*S*. *lissolab1atus*	20	2	H3		
*S*. *lissolab1atus*	21	14	H3/H32/H33		
*S*. *lissolab1atus*	22	8	H34/H35/H36		
*S*. *lissolab1atus*	23	20	H37		
*S*. *lissolab1atus*	24	2	H2		
*S*. *lissolab1atus*	25	15	H1		
*S*. *lissolab1atus*	26	20	H38		
*S*. *nukiangensis*	1	4	H9		
*S*. *nukiangensis*	2	4	H10		
*S*. *nukiangensis*	4	8	H6/H8/H16/H18/H21	R2	R1/R2/R3
*S*. *nukiangensis*	6	29	H6/H8/H11/H12/H13/H15/H18/H19	R2	R1/R2/R3
*S*. *nukiangensis*	7	16	H6/H8/H13/H15/H26/H28	R2/R3	R1/R2/R3
*S*. *nukiangensis*	8	38	H6/H8/H9/H12/H13/H14/H17/H18/H22	R11/R12	R1/R2/R3
*S*. *nukiangensis*	9	4	H14/H17/H18/H30	R1	R1
*S*. *nukiangensis*	10	36	H6/H8/H9/H13/H14/H17/H18/H20/H22/H27	R12	R1
*S*. *nukiangensis*	11	5	H6/H24	R2/R12	R1/R2
*S*. *nukiangensis*	12	59	H6/H8/H9/H18/H20/H22/H23/H24/H25/H29	R13	R1/R2/R3
*S*. *nukiangensis*	13	30	H6/H18/H20/H23/H24/H29/H31	R2	R1/R2/R3/R12

We obtained partial sequences for the recombinase-activating gene proteins 1 and 2 (*RAG*-*1* and *RAG*-*2*) from a subset of all samples (Table S1). The dataset included 75 sequences (1466 bp) from *RAG*-*1* and 61 sequences (1226 bp) from *RAG*-*2*. The longest non-recombining regions of *RAG*-*1* (1466 bp) and *RAG*-*2* (1226 bp) contained 16 and 15 variable sites, respectively.

### Phylogenetic relationship, mtDNA haplotype and nDNA allele relationships

The phylogenetic trees of the in-group obtained using *Cytb* via the Bayesian inference (BI) and maximum parsimony (MP) approaches showed a marked consistency in topological congruence, differing only in the support values for certain nodes; thus, only the MP tree was presented (Figure S2). The trees showed that *S*. *gongshanensis*, *S*. *lissolabiatus*, and *S*. *nukiangensis* were clustered into a single lineage, and *S*. *yunnanensis paoshanensis* generated another lineage (Figure S2). Thus, *S*. *yunnanensis paoshanensis* was excluded in the subsequent analyses.

**Figure 2:**
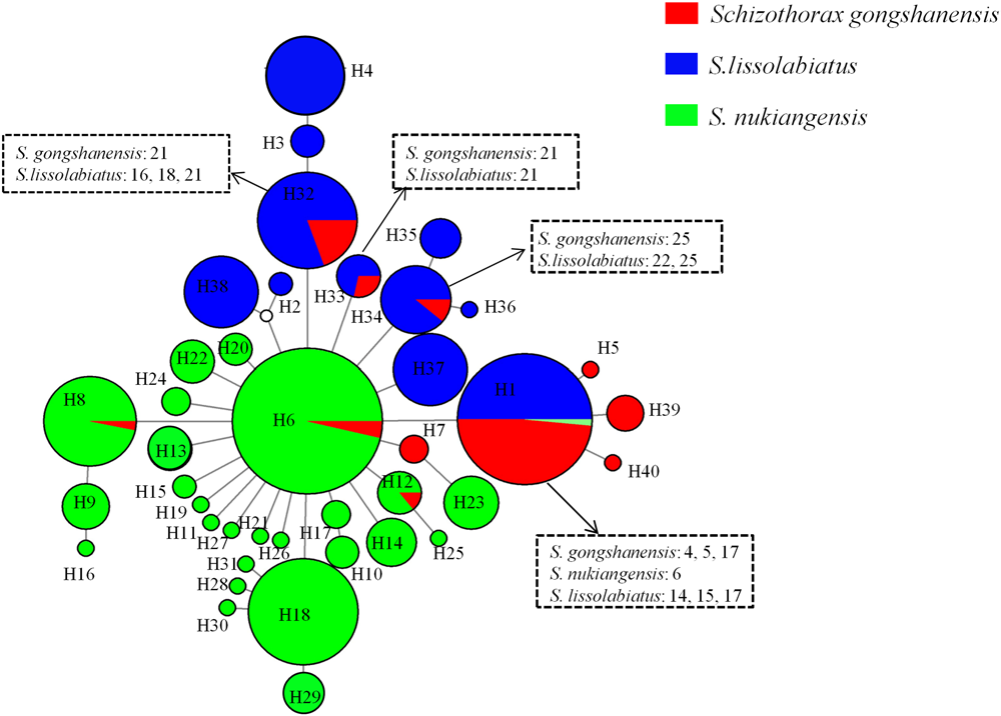
Median-joining network of *Cytb* for the *Schizothorax* species complex. Each colored circle represents different species, scaled according to its frequency in the entire sample. An empty circle indicates missing intermediate steps between observed haplotypes. The dotted rectangles indicate the populations of the three species with a shared *Cytb* haplotype. The location codes correspond to those in Table S1.

The grouping patterns of the *Cytb* median-joining network (MJN) clearly showed the haplotype relationships among the different species (Fig. 2). Only one haplotype (H1) was shared among the three species (including 32 *S*. *gongshanensis* specimens derived from the main stem, one *S*. *gongshanensis* specimen sampled from the tributary, 32 *S*. *lissolabiatus* specimens and one *S*. *nukiangensis* specimen). Four haplotypes (H1, H6, H8, H12) were shared between *S*. *nukiangensis* and *S*. *gongshanensis* (including 38 *S*. *gongshanensis* individuals and 115 *S*. *nukiangensis* individuals). Four haplotypes (H1, H32**–**H34) were shared by *S*. *gongshanensis* and *S*. *lissolabiatus*, and only one haplotype (H1) was shared by *S*. *lissolabiatus* and *S*. *nukiangensis*. From a total of 40 haplotypes, four haplotypes are private to *S*. *gongshanensis*, seven haplotypes are private to *S*. *lissolabiatus*, and twenty-two haplotypes are private to *S*. *nukiangensis*.

**Figure 3:**
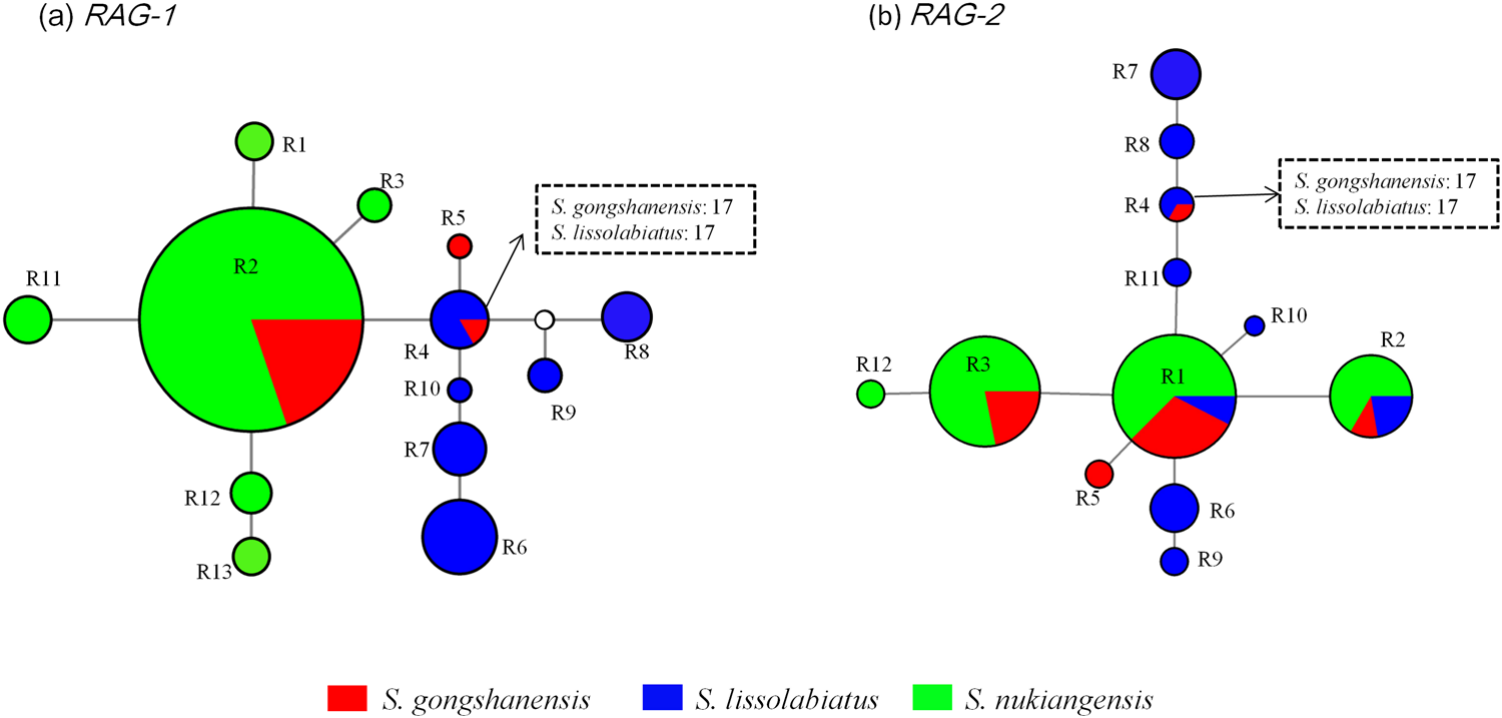
Median-joining network of the nuclear gene fragments for the *Schizothorax* species complex. Each colored circle represents different species, scaled according to its frequency in the entire sample. The dotted rectangles indicate the populations of the three species with a shared allele. An empty circle indicates missing intermediate steps between observed alleles. (**a**) *RAG-1*. (**b**) *RAG-2*.

We built MJNs using only the longest non-recombining regions of *RAG*-*1* and *RAG*-*2*. This strategy resulted in 13 and 12 alleles from *RAG*-*1* and *RAG*-*2*, respectively (Table 1). The networks obtained for *RAG*-*1* and *RAG*-*2* exhibited slightly different results (Fig. 3). The *RAG*-*1* MJN showed that *S*. *gongshanensis* from the main stem did share a particular allele with *S*. *nukiangensis*, and no shared alleles existed between the tributary populations (overall *S*. *lissolabiatus* specimens and *S*. *gongshanensis* from location 17) and the main stem populations (overall *S*. *nukiangensis* specimens and *S*. *gongshanensis* from the main stem) (Fig. 3a). In contrast, two mixed alleles were shared between the three species (*S*. *gongshanensis*, *S*. *lissolabiatus* and *S*. *nukiangensis*), and one mixed allele was shared between *S*. *gongshanensis* and *S*. *nukiangensis*, from the *RAG*-*2* MJN (Fig. 3b). In the both MJNs, both *S*. *gongshanensis* and *S*. *lissolabiatus* from the tritutary were found to share an allele.

### Genetic diversity and genetic differentiation

We calculated the haplotype and nucleotide diversities of the three species using *Cytb* sequences (Table S2). The haplotype and nucleotide diversities were greatest in *S*. *lissolabiatus* (0.863 ± 0.010 and 0.0028 ± 0.0001, respectively) and lowest in *S*. *gongshanensis* (0.676 ± 0.084 and 0.0014 ± 0.0002, respectively).

Pairwise comparisons of the genetic differentiation (ɸ_ST_) revealed significant genetic differentiation between the species pairs (Table 2). Low but statistically significant differentiation (P < 0.001) was determined for the three species based on *Cytb*. The analysis of nDNA genes showed that most of the pairwise species comparisons exhibited significant genetic differentiation (except between *S*. *gongshanensis* and *S*. *nukiangensis* from *RAG*-*2*) (Table 2). Furthermore, in the hierarchical analysis of molecular variance (AMOVA) results, we examined a low but significant genetic differentiation among the three species (ɸ_CT_ = 0.05, P = 0.048) (Table 3).

**Table 2:** Pairwise ɸ_ST_ values among the three species based on *Cytb* and two nDNA genes. The values in bold are significant at P < 0.05.

	ɸ_ST_		
	*Cytb*	*RAG-1*	*RAG-2*
*S*. *gongshanensis* vs. *S*. *lissolabiatus*	**0.131**	**0.358**	**0.203**
*S*. *gongshanensis* vs. *S*. *nukiangensis*	**0.256**	**0.153**	0.024
*S*. *lissolabiatus* vs. *S*. *nukiangensis*	**0.156**	**0.568**	**0.317**

**Table 3:** Results of AMOVA grouped by the three species based on *Cytb* sequences.

Source of variation	Percentage of variation	F_CT_	P
Grouped by species			
Among species	5.05	0.05	0.048
Among populations, within species	57.90	0.58	<0.001
Within populations	37.05	0.37	<0.001

We examined the pairwise genetic differentiation among populations with more than five specimens within each species (Tables 4, S3 and S4). For *S*. *gongshanensis*, low genetic differentiation was obtained between the two main stem populations, whereas moderate and significant differentiation was detected between the tributary population and the two main stem populations (Table S3). High and statistically significant differentiation (P < 0.05) was observed among most of populations within *S*. *lissolabiatus* (Table 4). With regard to *S*. *nukiangensis*, moderate and statistically significant differentiation was found between SJK population and other populations, whereas low genetic differentiation values were examined among the remaining populations (Table S4).

**Table 4:** Pairwise ɸ_ST_ values among the *S*. *lissolabiatus* populations based on *Cytb*.

	WQR	MAR	MKR	KGR	SCR	WDR	WMR	DSR
WQR								
MAR	0.000							
MKR	**1.000**	**1.000**						
KGR	**1.000**	**1.000**	**1.000**					
SCR	**0.604**	**0.597**	**0.241**	**0.675**				
WDR	**0.925**	**0.922**	**0.933**	**0.962**	**0.575**			
WMR	**1.000**	**1.000**	**1.000**	**1.000**	**0.623**	**0.933**		
DSR	**1.000**	**1.000**	**1.000**	**1.000**	**0.604**	**0.730**	**1.000**	
HDR	**1.000**	**1.000**	**1.000**	**1.000**	**0.820**	**0.968**	**1.000**	**1.000**

The values in bold are significant at P < 0.05. The location abbreviations are presented in Table S1.

### Gene flow

**Figure 4:**
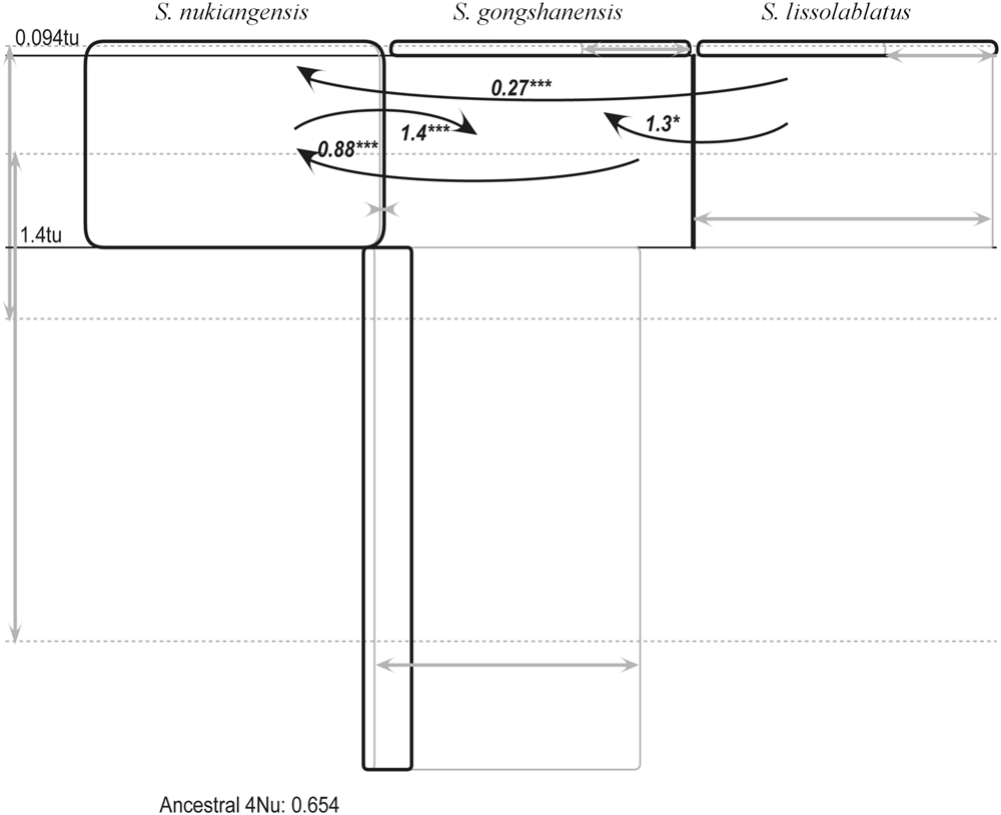
Isolation with migration analyses for the three *Schizothorax* species based on *Cytb*. The arrows represent migration directions from the source population to the receiving population; the numbers next to arrows are 2NM values. Only statistically significant cases of gene flow are presented. ^*^P < 0.05 and ^***^P < 0.001.

Simulation with IMa2 revealed statistically significant (P < 0.001) migration events among the three species (Fig. 4). Migration events occurred in both directions between *S*. *gongshanensis* and *S*. *nukiangensis* (2NM = 0.88 from *S*. *gongshanensis* to *S*. *nukiangensis* and 2NM = 1.4 from *S*. *nukiangensis* to *S*. *gongshanensis*), while migration events were unidirectional from *S*. *lissolabiatus* to *S*. *gongshanensis* (2NM = 1.3) and from *S*. *lissolabiatus* to *S*. *nukiangensis* (2NM = 0.27). The MDIV analyses showed that migration rate (*M*) was ranged from 2NM = 0.18 between *S*. *lissolabiatus* and *S*. *nukiangensis* to 2NM = 0.90 between *S*. *gongshanensis* and *S*. *lissolabiatus* (Table 5). Both analyses showed relatively low level of gene flow betweent *S*. *lissolabiatus* to *S*. *nukiangensis*.

**Table 5:** Gene flow and estimates of divergence times (in Ma) between species-pairs inferred from MDIV analyses.

Species-pair	*T*_*MRCA*_	*T*_pop_	M	θ	T_div_
*S*. *gongshanensis* vs. *S*. *lissolabiatus*	2.403	0.14	0.90	2.448	0.008
*S*. *gongshanensis* vs. *S*. *nukiangensis*	1.366	0.30	0.66	4.780	0.033
*S*. *lissolabiatus* vs. *S*. *nukiangensis*	1.562	0.58	0.18	4.550	0.061

*T*_MRCA_ and *T*pop are measured in units of 2*N*_e_θ; θ = 2*N*_e_μ, and μ is the mutation rate per sequence per generation. M and T_div_ indicate migration rates (2NM) and divergence times between species-pairs, respectively.

### Divergence time estimation

Net average sequence distances between species varied from 0.0004 to 0.0005 and yielded estimates of divergence between *S*. *gongshanensis*, *S*. *lissolabiatus* and *S*. *nukiangensis* dating to 0.02–0.025 Ma (Table S5). The MDIV results indicated that the divergence time between *S*. *gongshanensis* and *S*. *lissolabiatus* (0.008 Ma) was more recent than the divergence time between *S*. *gongshanensis* and *S*. *nukiangensis* (0.033 Ma) and between *S*. *lissolabiatus* and *S*. *nukiangensis* (0.061 Ma) (Table 5).

### Demographic history

Neutrality tests yielded statistically significant negative values for *S*. *gongshanensis* and *S*. *nukiangensis* (Table S2). The sum of square deviations and raggedness index suggested that the curves did not significantly deviate from the distributions expected under a model of sudden demographic expansion for *S*. *gongshanensis* (Table S2). The extended Bayesian skyline plot (EBSP) obtained similar results with the neutrality tests (Figure S3). EBSP suggested that population expansion for *S*. *gongshanensis* occurred between 0.06 Ma and 0.01 Ma. By contrast, *S*. *nukiangensis* appeared to experience population expansion between 0.04 Ma and 0.01 Ma. No signal of recent population expansion for *S*. *lissolabiatus* was detected in our analyses (Table S2; Figure S3).

## Discussion

### Genetic differentation among the three species

Genetic differentiation among the three closely related species was observed in the current study, although the haplotypes of the three taxa cluster into a single unresolved clade^9, 19^, and even the three species share mtDNA and nDNA haplotypes. Several lines of evidence for the genetic differentiation were comfirmed in our genetic analyses. First, only one *Cytb* haplotype, no *RAG-1* allele, and two *RAG-2* alleles shared between *S*. *lissolabiatus* and *S*. *nukiangensis* indicated remarkable genetic differentiation and low level of gene flow between them. Second, relatively low but statistically significant pairwise genetic differentiation (ɸ_ST_) was consistently obtained among the three species based on mtDNA and nDNA markers (Table 2). The genetic differentiation of nDNA markers larger than *Cytb* gene might be largely due to the difference in sample size and the number of populations used for nDNA genes and *Cytb*. We used the same sample to calculate ɸ_CT_ values and found that the genetic differentiation of *Cytb* was larger than the nuclear genes, except *S*. *lissolabiatus* vs. *S*. *nukiangensis* in *RAG-1* (Table S6). The nuclear genes with degenerate base used to calculate ɸ_CT_ values were firstly resolved into two alleles, which could increase the number of alleles and variable sites. This factor also could influence the ɸ_CT_ calculations. Thirdly, a hierarchical AMOVA based on *Cytb* supported a low but significant genetic differentiation among the three species (ɸ_CT_ = 0.05, P = 0.048).

On the Tibetan Plateau, the maximum extent of glacier development occurred during the middle Pleistocene (0.5 Ma), while glacial retreat has occurred since 0.17 Ma^20–22^, in contrast to the European and North American ice sheets, with a maximum extent in the Last Glacial Maximum (0.023–0.018 Ma)^22–24^. The Tibetan Plateau entered the last glacial age at approximately 0.075 Ma, continuing until 0.01 Ma^25, 26^. Mutation rates estimated for the *Schizothorax* species complex in the upper Mekong River^19^ placed divergence times between the species from 0.02 to 0.025 Ma based on net average sequence distances and from 0.008 to 0.061 Ma based on MDIV. Both estimates of divergence time consistently pointed to their separation during the last glaciation on the Tibetan Plateau (0.075–0.01 Ma). Considering that estimated divergence times vary depending on the assumed substitution rate, we cautiously hypothesized that the Pleistocene glaciations on the Tibetan Plateau facilitated the differentiation of the *Schizothorax* species complex in the Nujiang River. A similar case was observed in the *Gymnocypris chilianensis* in the Hexi River system^27^. During the last glaciation (0.075–0.01 Ma)^25, 26^, the drier and cold weather might divide the ancestral population of the *Schizothorax* species complex and result in the three present species diverging in allopatry. Secondary contact between the three new species was established due to coming of the warmer (interstadial) episodes during the glaciations^25^. Each glacial period was composed of a series of alternating cooler (stadial) and warmer (interstadial) episodes^21, 28^. Population expansion of *S*. *gongshanensis* and *S*. *nukiangensis* during the late Pleistocene inferred from demographic analyses confirmed the potential events of secondary contact and effects of the late Pleistocene glaciations on the *Schizothorax* species complex. Furthermore, trophic alternation due to oligotrophic condition during the glaciations may also be a potential explanation for the divergence of the three species^17^. The three species are clearly morphologically distinct regarding the pattern of the lower lip and lower jaw and the number of gill rakers (Table S7), which are all strongly associated with the ability to process different food types in schizothoracine fish^29, 30^. The absence of food during the glaciations increased the possibility for food competition and facilitated forming the shape of trophically relevant structures. The significant morphological differences can be maintained by disruptive natural selection. A similar process was seen in the *Gymnocypris* species complex in Lake Sunmcuo^31^.

### Haplotype shared among the three species

The sharing of haplotypes between/among different species can be attributed to one of two main processes, i.e., retention of ancestral polymorphisms due to recent divergence and gene flow. In our study, high frequency of haplotype shared was examined between *S*. *gongshanensis* and *S*. *lissolabiatus*, and between *S*. *gongshanensis* and *S*. *nukiangensis* (Figs 2 and 3). Because the hybrid zone where the two species-paris meet is broad (i.e., *S*. *gongshanensis* and *S*. *nukiangensis* are sympatric in some tributary and *S*. *gongshanensis* and *S*. *nukiangensis* occupy the same distribution between Fugong in Yunnan and southern section in Tibet in main stem^11^), recent gene flow seems a likely explanation. Furthermore, IM and MDIV results also detected substantial gene flow between the two species-pairs (Table 4, Fig. 4). In contrast, limited haplotypes shared and allopatric distribution between *S*. *lissolabiatus* and *S*. *nukiangensis* suggests rare gene flow between them, though relatively low level of gene flow was observed from the IM and MDIV calculations. However, the detected levels of gene flow might not be high enough to prevent differentiation; a 2NM greater than one would limit the divergence process in the absence of selection^32^. Consequently, shared ancestral polymorphisms is more suitable to interpret the haplotype sharing between the two species.

### Low genetic diversity and fragmentation of *S*. *lissolabiatus* populations

Genetic analyses indicated that *S*. *lissolabiatus* populations (locations 14–26) displayed remarkably low mtDNA genetic diversity. Seven out of nine populations with more than five individuals were found to harbor a single *Cytb* haplotype, except locations 21 and 22, which contained three *Cytb* haplotypes (Table 1). In addition, higher pairwise population differentiation and limited *Cytb* haplotype sharing among different *S*. *lissolabiatus* populations suggested rare gene flow and fragmentation among the tributary populations (Table 1; Fig. 2). The tributary is pretty small and separated by numerous mountains. The isolated tributaries posed a natural barrier for gene exchange and might lead to inbreeding within population. The inbreding would make populations more and more homogeneous and lose much genetic diversity^33^. Considering that small and isolated populations are inherently more vulnerable to external environmental perturbations and chance fluctuations^34^, more attention should be paid to conserving these populations in the future.

## Conclusions

Our study assembles mtDNA and nDNA loci to assess the genetic differentiation among the *Schizothorax* species complex in the Nujiang River basin and to explore the possible driving forces for their differentiation through dense sampling. Genetic analyses indicate relatively low but pronounced genetic differentiation between the three species-pairs and support the hypothesis that the last glaciation on the Tibetan Plateau (0.075–0.01 Ma) may drive the divergence of the species complex. Gene flow may be contribute to the haplotype sharing between *S*. *gongshanensis* and *S*. *lissolabiatus*, and between *S*. *gongshanensis* and *S*. *nukiangensis*, whereas retention of ancestral polymorphisms seems to be a better alternative for the explantion of the haplotype sharing between *S*. *lissolabiatus* and *S*. *nukiangensis*. In addition, the *S*. *lissolabiatus* populations with low genetic diversity and habitat fragmentation should be paid more attention for the future protection.

## Methods and Materials

### Ethics statement

All experimental protocols were approved by the Ethics Committee of the Institute of Hydrobiology, Chinese Academy of Sciences. The policies were enacted according to Chinese Association for Laboratory Animal Sciences, and coordinated with the Institutional Animal Care and Use Committee (IACUC) protocols (http://iacuc.usc.edu/).

### Sample collection, laboratory techniques and molecular data

A total of 430 specimens of three *Schizothorax* species (*S*. *gongshanensis*, *S*. *lissolabiatus* and *S*. *nukiangensis*) were collected from the Nujiang River basin from 2007 to 2013 (Table S1). Fresh samples were assigned to species based on differential morphological characters in the field (Table S5). In addition, 17 published sequences of partial mitochondrial cytochrome *b* gene (*Cytb*) belonging to *S*. *gongshanensis*, *S*. *lissolabiatus* and *S*. *nukiangensis* were added to these analyses (Table S1). A small piece of white muscle tissue or fin was dissected from the right side of each specimen. All tissue samples used for genomic DNA extraction were preserved in 95% ethanol. A total of 26 sampling locations were considered in the present study (Fig. 1; Table S1). The sampling map was generated using the ArcGIS and modified in Microsoft Office.

Total genomic DNA was extracted from muscle or fin tissue samples by using a standard salt extraction method. A segment of the *Cytb* gene was amplified from all individuals using the universal primers L14724 and H15915^35^. The PCR conditions were identical for the partial *Cytb* gene, with an initial denaturation at 94 °C for 3 min, followed by 30 cycles of denaturation at 94 °C for 1 min, annealing at 59–64 °C for 1 min, extension at 72 °C for 1 min, and a final extension at 72 °C for 5 min. Gene fragments of recombinase-activating gene proteins 1 and 2 (*RAG*-*1* and *RAG*-*2*) were obtained from a subset of samples (75 individuals for *RAG*-*1* and 61 individuals for *RAG*-*2*) using previously published primer sequences^36, 37^. The amplification of genomic DNA was conducted with an initial denaturation at 94 °C for 3–5 min, followed by 30–35 cycles of denaturation at 94 °C for 30 s, annealing at 53–55 °C for 30 s, extension at 72 °C for 1.5 min and a final extension at 72 °C for 10 min. The PCR reaction contained approximately 100 ng of template DNA, 1 μl of each primer (10 pmol/μl), 3 μl of 10 × reaction buffer, 1.5 μl of dNTPs (2.5 mM each) and 2.0 U of Taq DNA polymerase in a total volume of 30 μl. The amplified fragments were purified by 1.0% low-melting agarose gel electrophoresis and sequenced with an ABI PRISM 3700 (Applied Biosystems, Foster City, California, USA) automatic DNA sequencer using the same primer pairs.

### Sequence analyses

The nucleotide sequences were initially edited using DNASTAR multiple package (DNASTAR. Inc., Madison, WI, USA), aligned using Muscle^38^ and subsequently optimized in MEGA version 6.0^39^. Nuclear gene sequences containing more than one ambiguous site were resolved using PHASE 2.1.1^40, 41^, for which input files were prepared using SEQPHASE^42^. Recombination tests to detect the longest non-recombining region for each locus were conducted using IMGC^43^. Identical haplotypes of both mtDNA sequences and phased nuclear gene sequences were collapsed using DNASP 5.10^44^.

### Phylogenetic analyses

Phylogentic relationships among the three *Schizothorax* species in the Nujiang River were reconstructed using Bayesian inference (BI) and maximum parsimony (MP) approaches for *Cytb*. Two species of genus *Gymnocypris*, *G*. *eckloni* and *G*. *przewalakii*, were selected as outgroups for *Cytb* sequences (Table S1). Four published *Cytb* sequences of *S*. *yunnanensis paoshanensis* from the Nujiang River basin were added to the phylogenetic analyses (Table S1). Nucleotide substitution models were selected using the Akaike information criterion in MRMODELTEST version 2.3^45^. The best-fit model was GTR + I for *Cytb*. The BI analyses were performed in MrBayes 3.1.2^46^. Four independent runs were performed for 20 million generations. The phylogenetic trees were sampled every 1000^th^ generation, which resulted in 20 000 trees, and the first 25% were discarded as burn-ins. The MP analyses were implemented in MEGA version 6.0. Nodal support values were estimated from 1000 nonparametric bootstrap replicates.

### MtDNA and nDNA network

We used NETWORK 4.6^47^ to construct a median-joining network (MJN) for *Cytb*, *RAG*-*1* and *RAG*-*2*, respectively. A network approach is the most appropriate method to examine intraspecific gene evolution in closely related species, particularly when few characters are available for phylogenetic analysis as a result of shallow levels of divergence^48^. For *Cytb*, we directly analyzed the datasets. For the two nuclear genes, we analyzed the longest non-recombining region generated using IMGC.

### Genetic differentiation

Genetic variation, including haplotype diversity (*h*) and nucleotide diversity (π)^49^ with standard errors, was calculated for *Cytb* using DNASP 5.10. Pairwise genetic differentiation (ɸ_ST_) (i.e., ɸ-statistics)^50–52^ was calculated for the different species using *Cytb* and two nDNA loci in ARLEQUIN 3.5^53^. Analysis of molecular variance (AMOVA) was performed in ARLEQUIN 3.5 using *Cytb* to evaluate genetic differentiation within and among species. A total of 1000 permutations were employed to estimate the corrected significance levels using ɸ_ST_ analyses and AMOVA.

### Divergence time estimation

Two approaches were used to estimate the divergence times among the three species using *Cytb* sequences. First, we used net avarage sequence distance between species to estimate the approximate divergence times among the species. Net avarage sequence distance was estimated with MEGA version 6.0 as *d*A = *d*XY − (*d*X + *d*Y)/2, where *d*XY is the net average distance between species X and Y, and *d*X and *d*Y are the mean intraspecific distances. Mutation rate for *Cytb* (2.04 × 10^−8^ substitutions per site per year^19^) was used to measure the approximate divergence times between species.

Second, we calculated divergence times among the three species with a nonequilibrium coalescence model that uses the variance in pairwise differences between *Cytb* sequences to generate estimates of divergence time independent of gene-migration rates between pairs of species^54^. We used Markov chain Monte Carlo simulations as implemented in the program MDIV^54^ to estimate θ = 2*N*_e_μ, where *N*_e_ is the effective population size and μ is the mutation rate per sequence per generation. The time since divergence is *t*_pop_ = *t*/*N*_e_, where *t* is the time since population divergence. *M* is the migration rate between populations, and *T*_MRCA_ is the time to the most recent common ancestors between pair-wise species. MCMC simulations were run for 4 × 10^7^ steps with the first 10% discarded as burn-in. The uniform prior distribution of maximum *M* and *t*_pop_ was set to 10 and 30, respectively. Divergence times in generations before present (*T*_pop_) between pairs of species were estimated with *T*_pop_ = [(*t*_pop_ × θ)/2 *K*]/μ, where μ is the mutation rate per site per generation and *K* is sequence length. MDIV was run for three replicates with different random seeds using the HKY model^55^. The mutation rate of 2.04 × 10^−8^ substitutions per site per year was also used in MDIV.

### Gene flow

To determine whether the haplotype sharing between the three species-pairs stems from recent gene flow or from the retention of ancestral polymorphisms, potential gene flow among the three species was estimated using the isolation with migration (IM) model with the program IMa2^32^ and using the Markov chain Monte Carlo simulations with the program MDIV. IM analysis can address nonequilibrium scenarios where haplotype sharing may result from retention of ancestral polymorphism in recently diverged lineages and potential ongoing gene flow^32^. We used *Cytb* sequences for the IM and MDIV analyses. MDIV analysis was run using the aforementioned parameter settings. The method estimates the density functions and posterior-probability densities of the IM model parameters using a Markov chain (MCMC) method^56^. The functions of the model parameters were first estimated in M-mode with one million generations, and the first 10% were discarded as burn-in. The MCMC run was repeated three times to confirm convergence. Using these functions, the marginal posterior distribution and the maximum-likelihood estimates of the demographic parameters were then estimated in the L-mode. The HKY model of the DNA substitution was employed for *Cytb* and 40 heated metropolis-coupled Markov chains were employed to assure convergence.

### Historical demography

We assessed demographic historical changes using three approaches. First, Tajima’s *D* ^57^ and Fu’s *Fs* ^58^ statistics were calculated using ARLEQUIN 3.5 to detect evidence of demographical expansions, with 1000 coalescent simulations. Second, mismatch distributions^59^ were calculated to infer the demographic history in ARLEQUIN 3.5. The two aforementioned methods used only *Cytb* sequences. Finally, an extended Bayesian skyline plot (EBSP) was implemented in BEAST v.1.6^60^ to reveal demographic changes over time under neutral evolution^61^. EBSP facilitates the inclusion of mitochondrial and nuclear loci in the same analysis. The EBSP was performed for the three species independently, applying an evolutionary rate of 2.04% per million years. The evolutionary rates for two nuDNA genes were estimated as a function of the *Cytb* evolutionary rate. A strict clock model was set as prior, 100 million generations were run for *S*. *gongshanensis* and *S*. *lissolabiatus*, and 50 million generations were run for *S*. *nukiangensis*. Convergence was assessed with TRACER v.1.5^62^.

### Availability of supporting data

The data set supporting the results of this article is available in the GenBank under KT034083–KT034091, KT034105–KT034119, KT034121–KT034122, KT034131, KT034133–KT034149, KT034153–KT034175, KT034189–KT034199, KT034201, KT034213–KT034225, KT034229–KT034238, KT034259–KT034275, KT034322–KT034358, KU255547–KU255584, KY801703 and KY801706 and provided as supplementary information.

## Electronic supplementary material

Supplementary Information

Dataset 1
