# Supplementary material for: Genetic differentiation of the Schizothorax species complex (Cyprinidae) in the Nujiang River (upper Salween)
Source: Sci Rep. 2017 Jul 20;7:5944. doi: 10.1038/s41598-017-06172-5 (PMC5519740; doi:10.1038/s41598-017-06172-5)

**Supplementary information:**

**Genetic differentiation of the *Schizothorax* species complex (Cyprinidae) in the Nujiang River (upper Salween)**

Weitao Chen^1,2^, Xingjian Yue^3^, and Shunping He^1*^

^1^The Key Laboratory of Aquatic Biodiversity and Conservation of Chinese Academy of Sciences, Institute of Hydrobiology, Chinese Academy of Sciences, Wuhan, Hubei, 430072, China

^2^Graduate School of Chinese Academy of Sciences, Beijing, 10001, China

^3^School of Life Sciences, Neijiang Normal University, Neijiang, Sichuan, 641100, China

^*^Correspondence: clad@ihb.ac.cn

Corresponding author Phone: +862768780430; E-mail addresses: [clad@ihb.ac.cn](mailto:clad@ihb.ac.cn).

**Table S1**: Detailed information for specimens and sequences used in the present study.

**Table S2**: Nucleotide polymorphisms of the three species. N, number of sequences; Nh, number of haplotypes; h, haplotype diversity; Ph, private haplotype; π, nucleotide diversity; sd, standard error; SSD, sum of squared distribution; Hri, Harpending’s raggedness index. *P < 0.05,** P < 0.005, and ***P < 0.0005.

**Table S3:** Pairwise ɸ_ST_ values among the *S. gongshanensis* populations based on *Cytb*. The values in bold are significant at P < 0.05. The location abbreviations are presented in Table S1.

**Table S4:** Pairwise ɸ_ST_ values among the *S. nukiangensis* populations based on *Cytb*. The values in bold are significant at P < 0.05. The location abbreviations are presented in Table S1.

**Table S5:** Estimates of divergence times (in Ma) between lineage-pairs using net avarage sequence distance. T_div_ indicates divergence times between species-pairs.

**Table S6:** Pairwise ɸ_ST_ values among the three species based on *Cytb* used the same sample as the two nDNA genes. The values in bold are significant at P < 0.05.

**Table S7**: Morphological characters and distributions of *S. gongshanensis*, *S.* *lissolabiatus*, and *S. nukiangensis*.

**Figure S1**: A map showing the distribution range of the three species in the Nujiang River and other drainages where *S.* *lissolabiatus* distribute. Red, blue and green circles indicate the distribution range of *S. gongshanensis*, *S.* *lissolabiatus*, and *S. nukiangensis*, respectively. Map was created in the ArcGIS version 10.1 and modified in Microsoft Office.

**Figure S2:** Phylogenetic trees from a maximum parsimony analysis for the *Schizothorax* species complex in the Nujiang River based on *Cytb* haplotypes. The numbers at the top are the bootstrap proportions from a maximum parsimony analysis and Bayesian posterior probabilities. Red, *S. gongshanensis*; blue, *S.* *lissolabiatus*; and green, *S. nukiangensis*. Baby blue bar indicated haplotype were shared by two or three species.

**Figure S3:** Extended Bayesian skyline plot (EBSP) analysis of the three *Schizothorax* species. (a) *S. gongshanensis*, (b) *S.* *lissolabiatus*, (c) *S. nukiangensis*. *x*-axis, time in millenniums of years (ka); *y*-axis, the product of effective population size and generation length in years.

Table S2

|  | N | Nh | Ph | H ± sd | π ± sd | Tajima’*D* | Fu’s *FS* | SSD | Hri |
| --- | --- | --- | --- | --- | --- | --- | --- | --- | --- |
| *S. gongshanensis* | 58 | 11 | 4 | 0.676 ± 0.064 | 0.0014 ± 0.0002 | -1.67* | -4.17* | 0.0095 | 0.0706 |
| *S.* *lissolabiatus* | 156 | 11 | 7 | 0.863 ± 0.010 | 0.0028 ± 0.0001 | 0.15 | 1.03 | 0.0630* | 0.2254*** |
| *S. nukiangensis* | 233 | 26 | 22 | 0.827 ± 0.017 | 0.0015 ± 0.0001 | -1.85** | -17.65*** | 0.0022* | 0.0672* |

Table S3

|  | GS | GSP |
| --- | --- | --- |
| GS |  |  |
| GSP | 0.027 |  |
| SCR | **0.592** | **0.565** |

Table S4

|  | GSB | GSP | FG | PH | LS | MK | XPT |
| --- | --- | --- | --- | --- | --- | --- | --- |
| GSB |  |  |  |  |  |  |  |
| GSP | 0.000 |  |  |  |  |  |  |
| FG | 0.012 | 0.015 |  |  |  |  |  |
| PH | 0.000 | 0.000 | **0.057** |  |  |  |  |
| LS | 0.039 | **0.041** | 0.028 | **0.074** |  |  |  |
| MK | 0.000 | 0.000 | 0.000 | 0.036 | 0.000 |  |  |
| XTP | 0.017 | **0.076** | **0.076** | **0.087** | **0.095** | 0.032 |  |
| SJK | **0.238** | **0.308** | **0. 291** | **0.314** | **0.313** | **0.282** | **0.078** |

Table S5

| Species-pair | Net divergence | T_div_ |
| --- | --- | --- |
| *S. gongshanensis*-*S.* *lissolabiatus* | 0.0004 | 0.02 |
| *S. gongshanensis*-*S. nukiangensis* | 0.0004 | 0.02 |
| *S.* *lissolabiatus-S. nukiangensis* | 0.0005 | 0.025 |

Table S6

|  | ɸ_ST_ | |
| --- | --- | --- |
|  | *Cytb-RAG-1* | *Cytb-RAG-2* |
| *S. gongshanensis* vs. *S.* *lissolabiatus* | **0.528** | **0.360** |
| *S. gongshanensis* vs. *S. nukiangensis* | **0.250** | **0.208** |
| *S.* *lissolabiatus* vs. *S. nukiangensis* | **0.381** | **0.460** |

Table S7

| Species | Distribution | Shape of the mouth  and lower jaw | Average outer rakers of the first gill | Average inner rakers of the first gill |
| --- | --- | --- | --- | --- |
| *S. gongshanensis* | Between Fugong in Yunnan and southern section in Tibet and minority in some tributaries in the middle Nujiang River | Mouth subsuperior and lack horny ridge at the inner margin of the lower jaw and discontinuous lower lip | 14-17 | 19-24 |
| *S. lissolabiatus* | Tributaries in middle Nujiang River, Langcang River, Red River and Upper Pearl River | Mouth superior and horny ridge at the inner margin of the lower jaw and discontinuous lower lip | 13-21 | 18-31 |
| *S. nukiangensis* | Main stem and some large tributaries in Tibet | Mouth superior, horny ridge at the inner margin of the lower jaw and continuous lower lip | 17-26 | 26-33 |

Figure S1


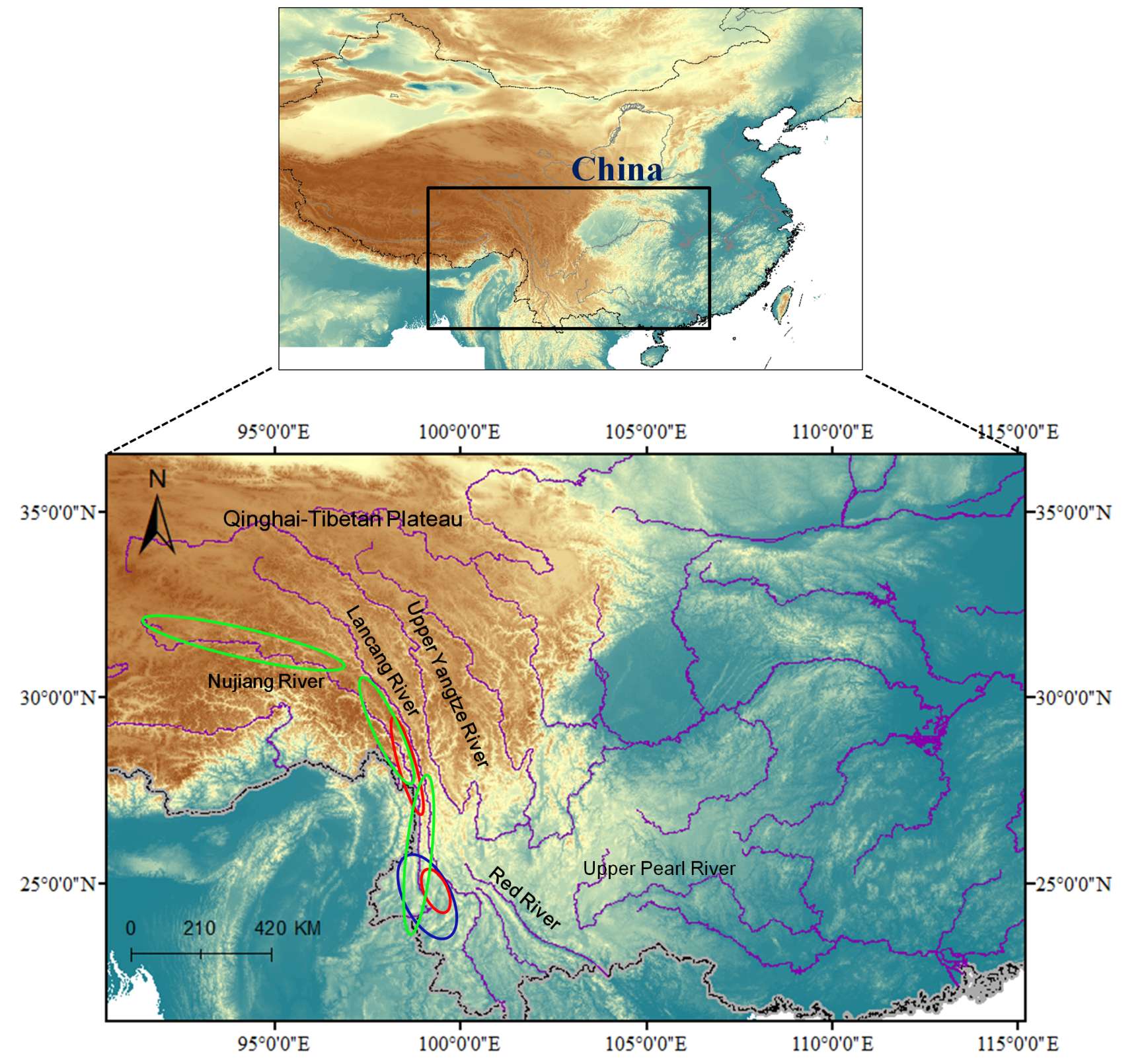


Figure S2


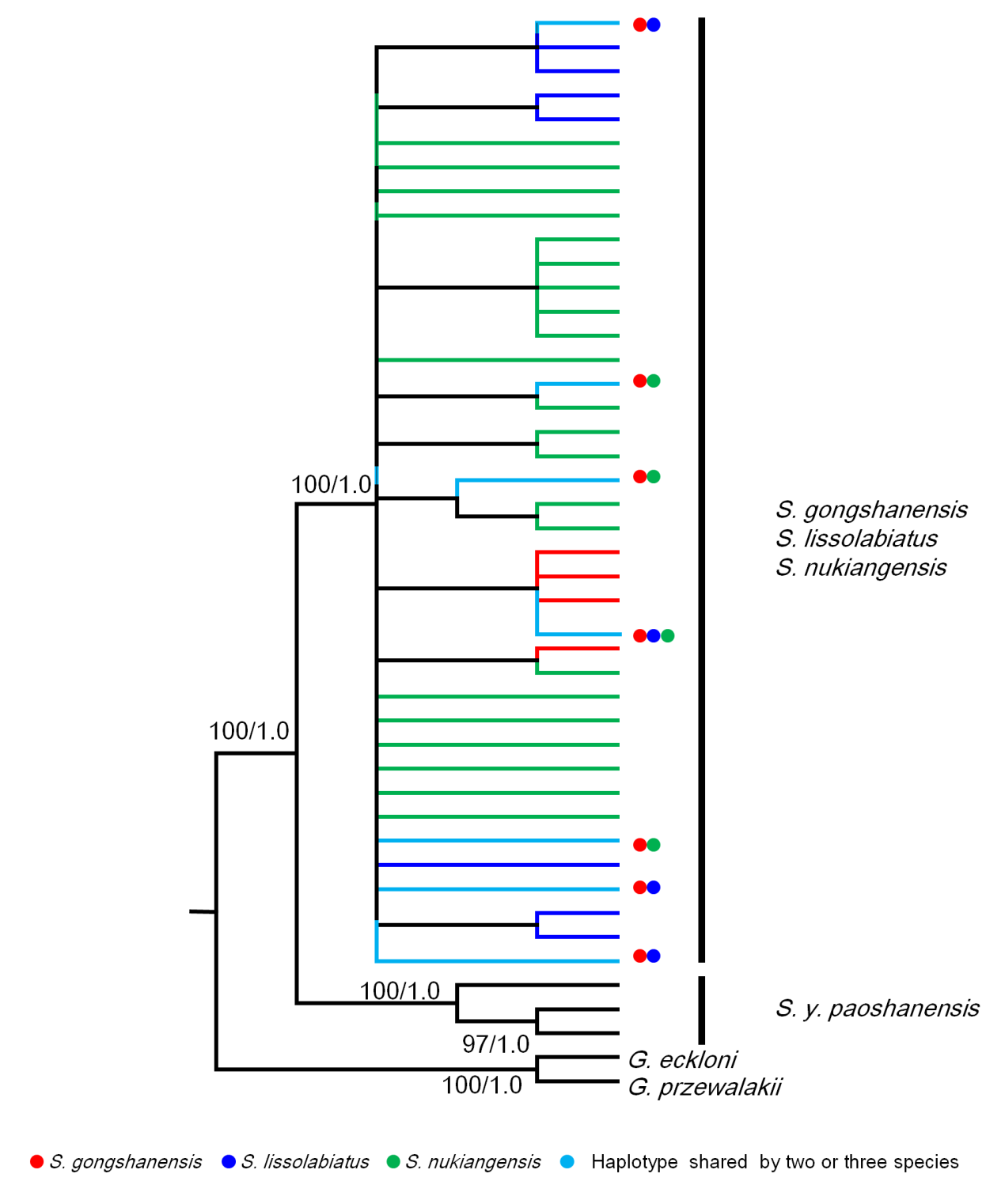


Figure S3


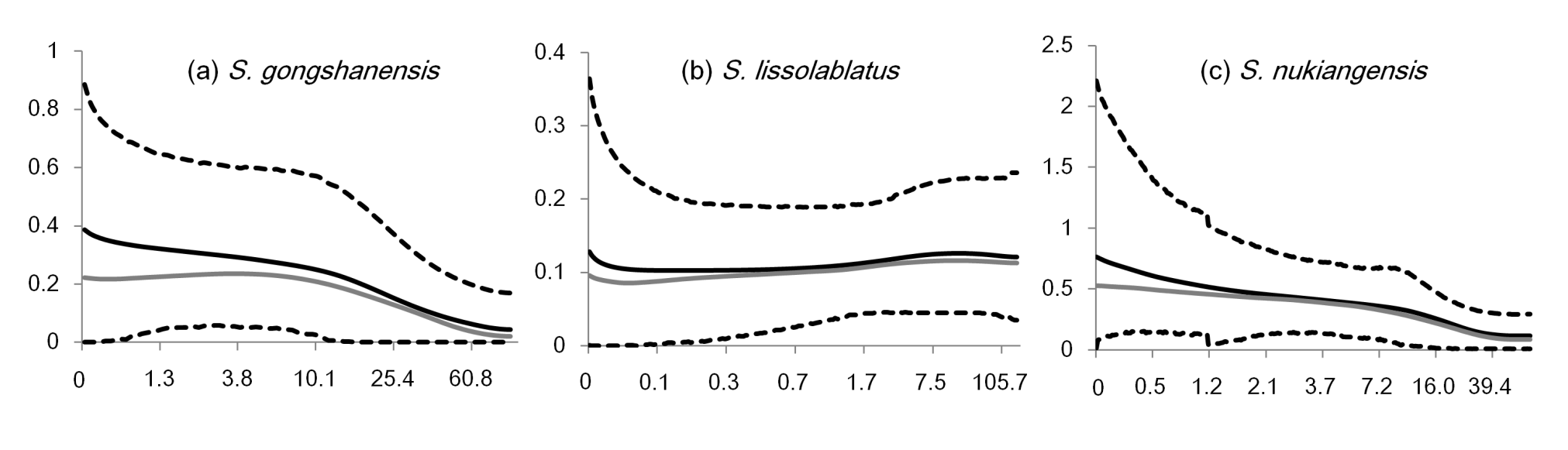

Supplement: Supplementary file 1 — Supplementary Information [file 41598_2017_6172_MOESM1_ESM.docx]
